# A Delayed Presentation of Late Dumping Syndrome After Ivor Lewis Procedure

**DOI:** 10.7759/cureus.40877

**Published:** 2023-06-23

**Authors:** Melanie Wong, James J Gome, Reinhardt Dreyer

**Affiliations:** 1 School of Medicine, Deakin University, Geelong, AUS; 2 General Medicine, Alfred Health, Melbourne, AUS; 3 Medicine, South West Healthcare, Warrnambool, AUS

**Keywords:** acarbose, glp-1, ivor lewis, gastric surgery, dumping syndrome

## Abstract

An accurate and timely diagnosis of dumping syndrome after gastric and oesophageal surgery is often difficult. A delay in making this diagnosis and instituting therapy can result in significant morbidity and avoidable complications. As bariatric surgery becomes more prevalent, the importance of a timely diagnosis of dumping syndrome is increasing. We present a case of a 77-year-old man who was admitted into the intensive care unit with a reduced conscious state secondary to hypoglycemia. The patient was subsequently diagnosed with late dumping syndrome in the context of an Ivor Lewis procedure seven years prior. Despite having a history of recurrent neuroglycopenic symptoms, there was a delay in diagnosis as dumping syndrome had not previously been considered until this admission. After confirmation of the diagnosis, the patient was commenced on dietary adjustments and acarbose, with a resolution of post-prandial hypoglycaemia. The authors discuss dietary and pharmacological therapy to manage hypoglycaemia associated with dumping syndrome.

## Introduction

Dumping syndrome is a post-surgical complication that is reported in up to 50% of patients following oesophagectomy and up to 75% of patients following Roux-en-Y gastric bypass and sleeve gastrectomy surgery [1,2]. Due to the anatomical alterations following these surgical procedures, gastric motility disturbances are a frequent complication. Dysmotility can lead to the rapid transition of undigested food to the small intestine and results in “early” or “late” dumping symptoms.

Early dumping symptoms occur within the first hour after ingestion and are attributed to rapid fluid shifts from intravascular compartments into the intestinal lumen containing hyper-osmolar food content. Gastrointestinal and vasomotor effects commonly described include bloating, abdominal pain, nausea, hypotension, tachycardia and syncope [1,3].

Late dumping symptoms occur 1-3 hours post-prandially due to excessive insulin release in response to a rapid carbohydrate load [2,3]. This results in hypoglycaemia with symptoms of diaphoresis, altered conscious state and tremor [4]. 

Dumping syndrome is diagnosed in patients with suggestive symptomatology after gastric or oesophageal surgery, with confirmation by provocation testing with an oral glucose tolerance test (OGTT) [1]. Dietary modification and pharmacological treatments including acarbose and octreotide have been proven effective [5,6].

## Case presentation

A 77-year-old man presented to a regional Victorian hospital with hypotension and hypoglycaemia, on a background of an Ivor Lewis procedure seven years prior for stage IA oesophageal adenocarcinoma.

On arrival, the patient presented with neuroglycopenic symptoms, a Glasgow Coma Score (GCS) of 8 out of 15, a blood glucose level (BGL) of 2.1 mmol/L and systolic blood pressure of 80 mmHg. There were no focal neurological signs apparent on admission. The neurological symptoms and hypoglycaemia resolved with oral glucose replacement. The patient required a brief period of inotropic support in the intensive care unit. Intravenous hydrocortisone was given with the concern of adrenal insufficiency and intravenous antibiotics for potential sepsis.

Following haemodynamic stabilisation, the patient reported episodes of post-prandial confusion at home for many years; he was unable to recall the exact year these symptoms began. The patient was hospitalised four years prior with an altered conscious state, expressive dysphasia and a BGL of 2.5 mmol/L. After full recovery, unremarkable brain imaging results and discussion with a stroke service, he was discharged with a presumed diagnosis of a transient ischaemic attack. On retrospective record review, the hypoglycaemia noted on admission was assumed a false reading after measuring a fasting BGL of 6.5 mmol/L. No further investigations or follow-up was arranged at the time.

Post-prandial hypoglycaemic episodes with associated neuroglycopenic symptoms were observed multiple times a day while undergoing hospital care. There was no preceding history of diabetes and no offending medications to explain the hypoglycaemia. No source of sepsis was identified and inflammatory markers remained within normal limits.

A computer tomography scan did not identify pancreatic or adrenal lesions. Early morning serum cortisol was 521 nmol/L (reference range: 145-619 nmol/L). A short Synacthen test demonstrated an appropriate cortisol response (pre-test cortisol: 326 nmol/L; 30 minutes post: 697 nmol/L; 60 minutes post: 764 nmol/L). At the time of a post-prandial hypoglycaemic event with a BGL of 2.8 mmol/L, his serum C-peptide level was 1.39 nmol/L (reference range: 0.30-1.30 nmol/L) and serum insulin level was 21 mIU/L (reference range: 3-25 mIU/L). Thyroid, liver and kidney function tests were normal and HbA1c was 5.2%.

An OGTT confirmed a diagnosis of late dumping syndrome with symptomatic hypoglycaemia 180 minutes after a 75 g glucose load (Table 1).

**Table 1:** Oral glucose tolerance test (75 g load)

Time	Plasma glucose (mmol/L)
0 minutes	5.1
60 minutes	10.0
120 minutes	4.4
180 minutes	2.6

The diagnosis of late dumping syndrome was based on 1) biochemical testing as above, 2) clinical symptoms of reactive post-prandial hypoglycaemia, 3) exclusion of autonomous insulin secretion and adrenal insufficiency, and 4) in the setting of a previous Ivor Lewis procedure.

Pharmacological management with acarbose (alpha-glucosidase inhibitor) 50 mg three times daily was commenced in combination with a low glycaemic index (GI) diet in divided meal portions. The patient was encouraged to abstain from alcohol intake as this was identified as a contributing factor to his reactive hypoglycaemia in the community. This resulted in the resolution of the post-prandial hypoglycaemia over the next 48 hours and no further neuroglycopenic episodes on review one month after discharge.

## Discussion

Dumping syndrome is primarily driven by rapid gastric emptying caused by post-operative gastric volume reduction, vagal nerve injury or pyloroplasty. This results in excess secretion of glucagon-like peptide 1 (GLP-1), an insulinotropic hormone, produced by intestinal L-cells in response to ingested simple carbohydrates [7]. Vasoactive gut hormones, such as neurotensin and vasoactive intestinal peptide, are also present in higher levels in dumping syndrome and contribute to vasodilation and hypotension [1]. Studies have shown lower rates of dumping syndrome in vagal-sparing oesophageal procedures [8].

Symptoms that aid a diagnosis of dumping syndrome include dizziness, diaphoresis, syncope and shock [9]. The case discussed in this report highlights that symptoms of dumping syndrome can be overlooked if not considered as a differential diagnosis. Dumping syndrome was not investigated during the hospital admission four years ago, and the post-prandial confusion the patient reported was likely neuroglycopenic in nature. Although the patient was unable to recall the exact duration of his symptoms, he likely had undiagnosed dumping syndrome for a prolonged period of time.

Diagnosis can be confirmed by an OGTT eliciting a higher plasma glucose level in the first 60 minutes, followed by hypoglycaemia 120-180 minutes after a glucose load. The cut-off value of 3.3 mmol/L has been suggested as a sensitive marker of post-prandial hypoglycaemia, although there is no definite value that has been widely accepted [6]. An OGTT has a sensitivity of 100% and a specificity of 94% for the diagnosis of dumping syndrome [2,10].

Dumping syndrome is best managed with dietary modification to prevent pathological GLP-1 production. This includes small-volume, divided meals that are low in GI and low in processed carbohydrates. Protein and fat intake should be optimised to supplement dietary needs. Alcohol should be avoided due to the risk of exacerbating hypoglycaemia by suppression of gluconeogenesis, and fluid intake should be postponed until at least 30 minutes after ingesting solid meals [6]. Lifestyle changes and dietician involvement are key to ensuring optimal outcomes. 

Pharmacological therapy should be considered in the case of severe symptoms or lack of improvement despite dietary modifications. Acarbose competitively inhibits alpha-glucosidase at the intestinal brush border primarily delaying the conversion of sucrose to glucose and fructose, as well as delaying the digestion of complex carbohydrates and subsequent rise in serum glucose levels [11]. The net effect of this is to reduce hypersecretion of insulin in response to a carbohydrate load, which underlies the pathophysiology of reactive hypoglycaemia. Studies have shown that 50 mg doses of acarbose three times a day after meals have reduced post-prandial hypoglycaemia [12]. There does not appear to be any additional benefit in using higher doses of acarbose [1]. The most common adverse effects are bloating and flatulence as a result of carbohydrate malabsorption [3,6]. There is insufficient evidence to define the optimum duration of treatment with acarbose for dumping syndrome.

In severe, refractory dumping syndrome, other pharmaceutical therapies can be considered. Diazoxide, a potassium channel activator, potentiates the potassium channels in pancreatic beta cells, which results in the reduction of insulin secretion. A multi-centre, retrospective, case series in 2016 showed a reduction in hypoglycaemic events in 50% of patients treated with diazoxide for late dumping syndrome after bariatric surgery [13]. Octreotide is a synthetic somatostatin analogue that has proven to effectively reduce insulin secretion and prevent post-prandial hypoglycaemia in up to 90% of those with refractory late dumping syndrome [1,10]. Adverse effects associated with octreotide are pain at local injection sites, diarrhoea, steatorrhea and gallstones. 

## Conclusions

Dumping syndrome is a complication following gastric and oesophageal surgery that should not be missed. As the field of bariatric surgery is expanding, the prevalence of dumping syndrome is likely to increase. This case highlights the severity of symptoms that can occur and the importance of early investigation and intervention to avoid serious consequences and hospitalisations.
